# Fenestration: Integrating Wings Into the Atrial Septal Occluder for Navigating a Challenging Terrain

**DOI:** 10.7759/cureus.45260

**Published:** 2023-09-14

**Authors:** Anil K Singhi, Soumya K Mohapatra, Dilip Kumar, Arindam Pande, Ashesh Halder, Somnath Dey, Anish Nath, Arnab De

**Affiliations:** 1 Pediatric and Congenital Heart Disease, Medica Superspecialty Hospital, Kolkata, IND; 2 Cardiology, Medica Institute of Cardiac Sciences, Medica Superspecialty Hospital, Kolkata, IND; 3 Cardiology, Medica Superspecialty Hospital, Kolkata, IND; 4 Cardiac Anaesthesia and Critical Care, Medica Superspecialty Hospital, Kolkata, IND

**Keywords:** duchenne muscular dystrophy, left atrial arrhythmia, systemic lupus erythematosus, pulmonary valve stenosis, pulmonary hypertension, diastolic dysfunction, fenestration, septal occluder, comorbidities, atrial septal defect

## Abstract

Background

Atrial septal defect (ASD) closure with significant left-to-right shunt and concurrent comorbidities poses challenges for intervention. A fenestrated atrial septal defect (FASD) device is a viable option for patients who cannot undergo complete occlusion due to hemodynamic and medical reasons. This study explores the use of FASD occluders in patients with secundum ASD and associated comorbidities where complete occlusion is difficult.

Methodology

This retrospective study collected the details of patients recommended for FASD closure diagnosed with significant secundum ASD and who had additional comorbidities between July 2015 and July 2023 in a tertiary cardiac center in eastern India. Among this cohort, patients who underwent FASD device placement were subjected to a comprehensive analysis.

Results

In total, 16 patients diagnosed with secundum ASD, characterized by significant left-to-right shunt and concurrent comorbidities, were considered for FASD closure during the study period. Ultimately, 13 patients (first group) underwent fenestrated atrial septal occluder implantation. The average age was 45.07 years, with the majority being females (n = 9). Comorbidities among this cohort included substantial left ventricular diastolic dysfunction (n = 7), left ventricular diastolic dysfunction coupled with moderate pulmonary hypertension (n = 1), severe pulmonary hypertension (n = 1), severe pulmonary valvular stenosis with right ventricular diastolic dysfunction (n = 2), and systemic lupus erythematosus (SLE) (n = 2). From this cohort, three patients did not undergo the intervention. The second group consisted of an elderly patient with severe left ventricular diastolic dysfunction, a young adult with a history of left atrial arrhythmia, and a child with Duchenne muscular dystrophy (DMD). The average ASD size among patients who underwent the intervention was 26.38 mm, with a thick-to-thick dimension measuring 31.15 mm. The procedure was successful in all 13 patients, with the most frequently used device being a 34 mm occluder (range = 28-40 mm). All devices, excluding the initial one, were custom-made atrial septal occluders (Lifetech Scientific). Among the patients, 12 exhibited left-to-right fenestration flow, while one patient experienced fenestration constriction, likely due to occluder overcrowding. The first patient had a handmade 5 mm fenestration in a 40 mm Amplatzer septal occluder, which got closed off at the one-year follow-up. The procedure was well-tolerated hemodynamically in all patients, with no major complications during the peri-procedural period. Short-term follow-up indicated favorable patient progress.

Conclusions

FASD closure emerges as a pivotal alternative for intricate scenarios involving secundum ASD coupled with concurrent comorbidities, offering individualized tailored solutions. Alongside the conventional associated comorbidities, such as left ventricular diastolic dysfunction and pulmonary hypertension, FASD devices hold the potential to extend their benefits to patients grappling with other complexities, including severe pulmonary valvular stenosis, SLE, predisposition to left atrial arrhythmia, and conditions like DMD. Ensuring meticulous evaluation of patient suitability and providing ongoing vigilant care becomes paramount for achieving optimal outcomes. The validation of these findings and the broadening of the comprehension of this approach necessitate further comprehensive investigations.

## Introduction

Atrial septal defects (ASDs) constitute approximately 10% of all congenital heart diseases. Closing the defect in the presence of a significant left-to-right shunt and operable hemodynamics is considered reasonable at any age [1]. The presence of additional medical conditions or diseases in a patient with secundum ASD, such as pulmonary hypertension, left ventricular diastolic dysfunction, and severe pulmonary valve stenosis, can impact the overall health of the patient and may require additional medical attention and management strategies. Fenestration involves creating an opening in the patch or device to allow controlled blood flow between the atria. This approach is tailored to patients with specific comorbidities that preclude standard ASD closure [2,3]. Cho et al. documented surgically fenestrated closures of ASDs in 16 patients, even in the presence of severe pulmonary hypertension. This approach, combined with tricuspid annuloplasty, yielded noteworthy results. Specifically, the study highlighted enhancements in functional classification in 81.2% (13 patients) and tricuspid regurgitation grade in 93.7% (15 patients) of the cases. These improvements were achieved without encountering significant complications [3]. The emergence of ASD occluders designed with purpose-built fenestrations has further intensified the appeal of transcatheter intervention for both patients and caregivers.

This study explores the use of fenestrated atrial septal defect (FASD) devices for patients with secundum ASDs who cannot undergo complete occlusion due to hemodynamic and medical reasons.

## Materials and methods

Study design and patient population

This retrospective study aimed to analyze patients with a significant secundum ASD and various additional comorbidities who were recommended for FASD closure between July 2015 and July 2023 at a tertiary care center in India. The demographic, clinical, and procedural characteristics, as well as the short-term outcomes of the cohort of patients who underwent the intervention, were analyzed in detail.

Ethical considerations

All patients provided informed consent for the procedure and subsequent data collection and analysis for research purposes per the hospital’s general consent protocol. Ethical approval was obtained from the institutional ethics committee before data collection and analysis. The institutional ethics committee protocol allowed waiver of the requirement for patient consent for this retrospective record-based study vide letter number CERC/2022/NOV/ 1(v). Patient information was anonymized to protect patient identity.

Data collection

Demographic data, including age, sex, and relevant comorbidities, were collected for each patient. The clinical details encompassed a comprehensive assessment of the patient’s medical history, including any relevant cardiac conditions and comorbidities. Additionally, indications for the FASD closure were documented for each patient, outlining the rationale behind recommending the intervention.

Procedure details

The patients in this study underwent a comprehensive examination that included a clinical evaluation, electrocardiogram, and chest X-ray. Transthoracic echocardiography and transesophageal echocardiography were performed to identify the anatomy of the defect, assess pulmonary artery pressure, and evaluate left ventricular diastolic function. The diastolic dysfunction of the left ventricle was assessed with the help of pulsed Doppler and tissue Doppler analysis of the mitral valve. If present, the mean gradient across the ASD was noted. A mean gradient of more than 2 mmHg across the ASD was considered significant. This was taken as one of the markers for left atrial hypertension and left ventricular diastolic dysfunction. All patients underwent cardiac catheterization and coronary angiography, where the right heart pressure and left ventricular end-diastolic pressure (LVEDP) were measured. Pulmonary blood flow and pulmonary vascular resistance were calculated for all patients. The systolic pulmonary artery pressure was used to classify the different grades of pulmonary hypertension, with mild pulmonary hypertension ranging from 35 to 50 mmHg, moderate ranging from 50 to 70 mmHg, and severe being greater than 70 mmHg [4]. The post-device closure pulmonary artery pressure was measured in patients with moderate-to-severe pulmonary arterial hypertension. An LVEDP of 15 mmHg and above was considered elevated [5]. Patients with left ventricular diastolic dysfunction underwent balloon occlusion of the ASD. Elevation of the LVEDP by 5 mm from the basal state was considered significant, indicating that the patient needed closure of the ASD with FASD [6].

Occluders

The fenestration of the first device was performed by making a handmade 5 mm opening on the 40 mm Amplatzer Septal Occluder (Abbott, Abbott Park, Illinois). The subsequent occluders were custom-made Lifetech fenestrated septal occluders (Lifetech Scientific, Shenzhen, China). The FASD occluder was custom-made by the company on request by providing the patient’s anatomical details and device requirements. The size of the FASD occluder was chosen based on the anatomy of the defect. The thick-to-thick dimension of the ASD was used for FASD selection. The custom-made fenestrated Lifetech atrial septal occluder had a 5 mm opening in the 22-24 mm occluders, a 6 mm opening in the 26-30 mm occluders, and an 8 mm opening in the occluders 32 mm and larger. The fenestration size was decided by the company on testing the stability and integrity of the structure.

Statistical analysis

The continuous variables are expressed as the mean ± standard deviation, and the categorical variables are expressed as the frequency and percentage. All data were analyzed using Microsoft Excel software (Microsoft Corp., Redmond, WA, USA).

## Results

Between July 2015 and July 2023, 16 patients with secundum ASD with significant left-to-right shunt and additional comorbidities were recommended for FASD closure. Of the 16 patients, three were excluded from receiving the intervention due to various causes. As a result, the final intervention group consisted of 13 patients (Table 1).

**Table 1:** Distribution of patients with ASD and concurrent comorbidities who were candidates for closure using the FASD device. ASD: atrial septal defect; FASD: fenestrated atrial septal defect; PVRI: pulmonary vascular resistance index

Patient categories with comorbidities	Number of patients
Total patients recommended for FASD closure	16
(A) Total patients undergoing FASD closure (first group)	13
(i) Left ventricular diastolic dysfunction	7
(ii) Left ventricular diastolic dysfunction and moderate pulmonary hypertension	1
(iii) Severe pulmonary hypertension (elevated PVRI >5 wU.m^2^)	1
(iv) Severe valvar pulmonary stenosis with right ventricular diastolic dysfunction	2
(v) Systemic lupus erythematosus	2
(B) Patients not undergoing FASD closure (second group)	3
(i) Elderly lady with ASD and significant left ventricular diastolic dysfunction	1
(ii) Adult patient with ASD and a history of left atrial arrhythmia	1
(iii) Child with ASD, Duchenne muscular dystrophy, and left ventricular diastolic dysfunction	1

The majority of the patients in the cohort were female (n = 9). The mean age of the patients in this study was 45.07 years (range = 11-64 years). The detailed demographic and hemodynamic parameters of the patients are presented in Table 2 and Table 3.

**Table 2:** Demographic details of the patients who successfully underwent fenestrated atrial septal defect device closure. ASD: atrial septal defect; SD: standard deviation; BPV: balloon pulmonary valvotomy; CAG: coronary angiography; FASD: fenestrated atrial septal defect device; PTCA: percutaneous transcatheter coronary angioplasty; PVRI: pulmonary vascular resistance index

Parameter	Value
Number of patients	13
Male:Female	4:9
Age in years, mean ± SD (range)	(45.07 ± 14.4), (11–64)
Weight in kg (mean ± SD)	49.22 ± 11.7
ASD size in mm (mean ± SD)	26.38 ± 5.09
ASD size (thick to thick) in mm (mean ± SD)	31.15 ± 4.49
Median device size in mm (range)	34 (28–40)
Amplatzer septal occluder, handmade fenestration	n = 1
Lifetech septal occluder with custom-made fenestration	n = 12
FASD closure during the initial procedure	n = 9
FASD closure as the second procedure	n = 4
(A) BPV and FASD closure	2 (on different day one, same day one)
(B) Coarctation stenting and FASD closure	1 (on different day)
(C) PTCA and FASD closure	1 (on different day)
Fluoroscopy time in minutes, mean ± SD	13.46 ± 6.26
Follow-up duration in months, mean ± SD (range)	6.03 ± 4.66 (1–16.5 months)

**Table 3:** Clinical parameters and follow-up data. LVEDP: left ventricular end-diastolic pressure; SD: standard deviation; PASP: pulmonary artery systolic pressure; Qp:Qs: pulmonary to systemic blood flow ratio; PVRI: pulmonary vascular resistance index; wu.m^2^: wood unit.m^2^

Parameter	Value
Saturation at the basal state	Average = 97.8% (range = 90–100%)
Saturation at the time of follow-up	Range = 97–100%
Functional class at the basal state	II (n = 11), III (n = 2)
Functional class at the time of follow-up	I (n = 12), II (n = 1)
LVEDP mmHg	16.75 ± 4 (mean ± SD)
Qp:Qs	2.10 ± 0.58 (mean ± SD)
Pulmonary artery mean pressure, mmHg	27 ± 10.8 (mean ± SD)
Normal Pulmonary artery pressure, PASP <35 mmHg	n = 6
Mild pulmonary artery hypertension, PASP: 35–50 mmHg	n = 4
Moderate pulmonary artery hypertension, PASP: 50–70 mmHg	n = 2
Severe pulmonary artery hypertension, PASP >70 mmHg	n = 1
Baseline PVRI (wu.m^2^)	2.67 ± 1.92 (mean ± SD)
PVRI <2 wu.m^2^	n = 7
PVRI 2–5 wu.m^2^	n = 5
PVRI >5 wu.m^2^	n = 1 ( value- 8.04 wu.m^2^)
Good fenestration flow in follow-up	n = 11
Reduced fenestration flow in follow-up	n = 1 (oversized device)
Fenestration closed in follow-up	n = 1 (at one year, handmade fenestration)
Coronary angiogram: normal, no atherosclerotic disease	n = 11
Coronary angiogram, post-stenting for double-vessel disease	n = 1, good flow
Coronary angiogram not done	n = 1 (an 11-year-old child)
Aspirin	n = 13
Clopidogrel	Total patient = 4; large device (n = 3); post-coronary stenting (n = 1)
Warfarin	n = 1 ( pulmonary hypertension)

**Figure 1:**
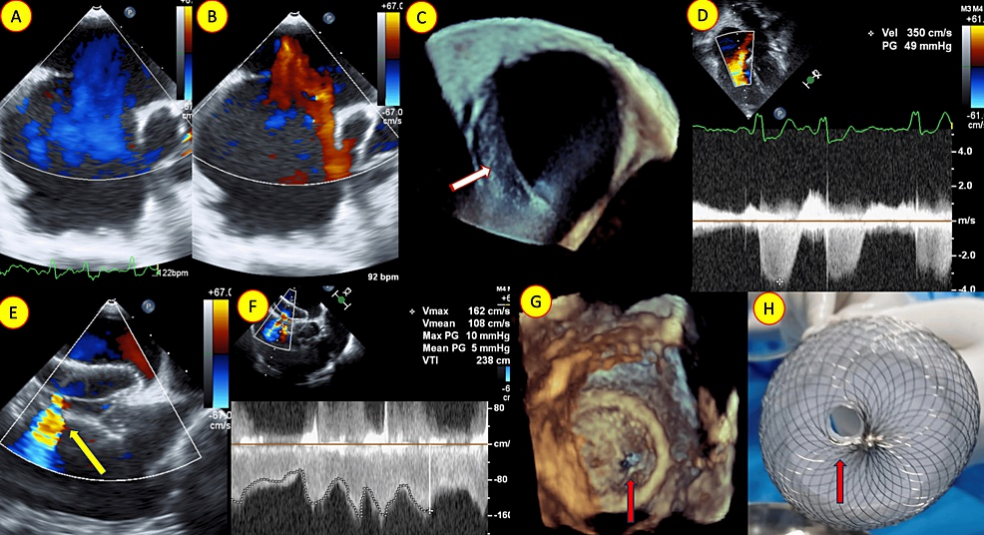
Transesophageal echocardiogram (TEE) in zero-degree view with color Doppler showing (A) left-to-right and (B) right-to-left shunt. (C) Three-dimensional enface TEE view showing the secundum atrial septal defect (ASD) with a thin floppy posterior inferior margin (white arrow). (D) The tricuspid regurgitation gradient is seen in the apical four-chamber view. (E) TEE in 90-degree view showing the atrial septal occluder in a stable position and the custom-made fenestration (yellow arrow) flowing left to right. (F) The continuous wave Doppler of the fenestration flow had a mean gradient of 5 mmHg. (G) Transesophageal three-dimensional enface view from the right atrial side showing the atrial septal occluder with the fenestration (red arrow). (H) The custom-made fenestration (red arrow) in the 40 mm Lifetech atrial septal occluder is shown.

The majority of the patients were in the functional class II with two in the functional class III. The average saturation was 97.8% (range = 90-100%). One of the patients had a baseline desaturation of 90%, which was attributed to bidirectional shunt across the ASD, moderate pulmonary hypertension, and associated chronic obstructive lung disease (COPD). The saturation improved to 95% after COPD treatment by the pulmonologist. During the cardiac catheterization of this patient, the left-to-right shunt ratio was calculated as 3.1. The pulmonary artery systolic pressure was 46 mmHg and the mean pressure was 31 mmHg. The LVEDP at baseline was 18 mmHg. The ASD was closed with a 40 mm FASD device (Figure 1).

**Figure 2:**
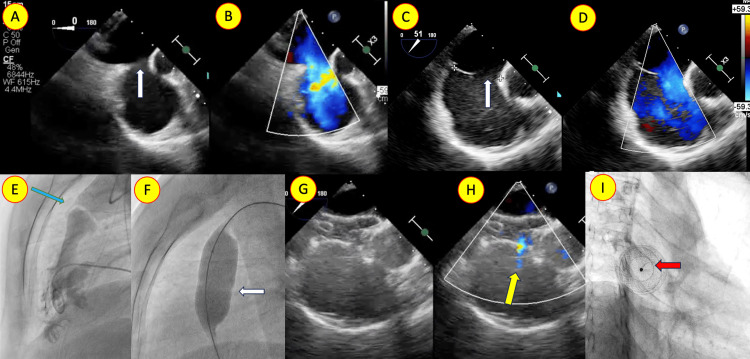
Transesophageal echocardiogram (TEE) in zero-degree view (A, B) and 50-degree view (C, D) with color Doppler showing the moderate secundum atrial septal defect (ASD) (white arrow) with absent aortic rim and thin floppy posterior rim. Angiogram in the lateral view showing (E) doming of the pulmonary valve (blue arrow) and disappearance of the waist during balloon dilatation (white arrow). (G) TEE after device closure with color Doppler (G, H) showing the stable ASD device and fenestration flowing left to right (yellow arrow). (H) Fluoroscopic image showing the fenestration (red arrow) in the oblique view.

The mean ASD size of the cohort was 26.38 mm, with a thick-to-thick measurement of 31.15 mm. The thick-to-thick measurement of the ASD was used for deciding the size of the occluder. The predominant indication of FASD closure was left ventricular diastolic dysfunction (n = 7), and one had left ventricular diastolic dysfunction with moderate pulmonary hypertension. Two patients had associated severe valvular pulmonary stenosis, with one having a bidirectional shunt across the ASD in the basal state and the other having a significant valvular pulmonary stenosis, with the shunting across the ASD predominantly being left-to-right with evidence of bi-directionality. The right ventricular end-diastolic pressure was elevated in both of these patients. The first patient had a balloon pulmonary valvotomy initially, followed by FASD closure at a later date. The second patient underwent both procedures on the same day (Figure 2).

Additionally, one patient had ASD and severe pulmonary hypertension with elevated pulmonary vascular resistance, and two had systemic lupus erythematosus (SLE). One of the SLE patients had mild pulmonary hypertension, and the second had normal pulmonary pressure. The fenestrated devices in the SLE patients were preemptively used to aid in the probable future occurrence of pulmonary arterial hypertension. The SLE patients continued to be on therapy for SLE under rheumatology care.

In addition, three patients who had been recommended for FASD closure due to associated comorbidities did not undergo the procedure for various reasons (Table 1) and were excluded from this analysis. The first patient was an elderly female with ASD and significant left ventricular diastolic dysfunction. The patient experienced significant respiratory distress in the context of COPD and chronic renal failure. After recovering from this episode, the patient and their family members engaged in discussions regarding the treatment plan with the attending physician, an intensive care specialist, a cardiologist, and a nephrologist. They opted to forgo the intervention and instead continue with medical follow-up. The second patient was a young adult with a history of intermittent left atrial arrhythmia. The patient was recommended for FASD closure with the possibility of requiring an electrophysiological study and radiofrequency ablation. The patient decided to undergo the treatment elsewhere for personal reasons. The third patient was a young child with ASD, Duchenne muscular dystrophy (DMD), and left ventricular mild thickening with diastolic dysfunction. The parents expressed the desire to defer the intervention until after the child received treatment for DMD.

**Figure 3:**
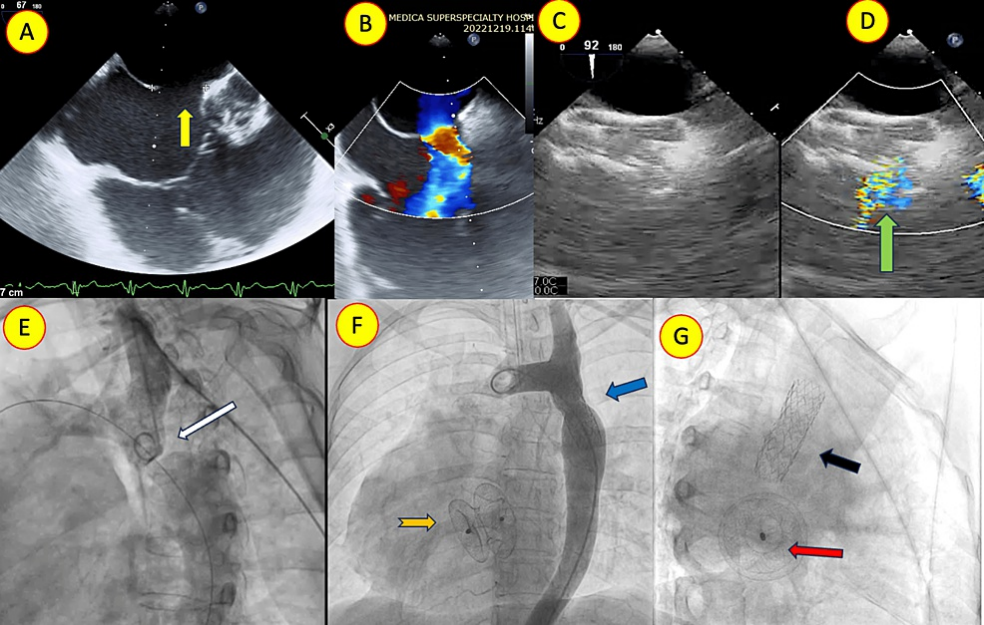
Transesophageal echocardiogram (TEE) with color Doppler (A, B) showing the moderate secundum atrial septal defect (ASD) (yellow arrow) with an absent aortic rim and floppy posterior rim. (G) TEE after device closure with color Doppler (C, D) showing the stable ASD device and fenestration flowing left to right (green arrow). (E) Aortic angiogram in the oblique view showing severe post-subclavian coarctation of the aorta (white arrow). (F) Two-year follow-up aortogram after the ASD device (orange arrow) closure showing good stent flow (blue arrow). (H) Fluoroscopic image showing the fenestration (red arrow) in the oblique view. The good aortic stent position is also seen (black arrow).

FASD closure was done during the initial procedure in nine patients, while in four, it was performed as a second procedure (Table 2). One patient had severe coarctation of the aorta, hypertension, and significant left ventricular diastolic dysfunction. The patient underwent coarctation of the aorta stenting first, followed by FASD closure after a few months (Figure 3).

**Figure 4:**
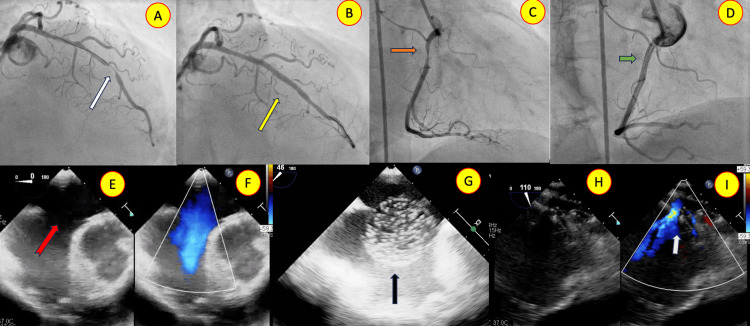
(A) Left coronary angiogram showing tight obstruction of the left anterior descending artery (white arrow), (B) post-stenting good flow across the narrow segment is seen (yellow arrow). (C) Right coronary artery angiogram showing tight narrowing (orange arrow), (D) good post-stenting flow is visualized (green arrow). (E, F) Transesophageal echocardiogram (TEE) with color Doppler showing the secundum atrial septal defect (red arrow). (G) Balloon (black arrow) occlusion was done to demonstrate the left ventricular diastolic pressure change. (H, I) TEE with color Doppler post-fenestrated device closure showing the stable device position and fenestration flowing left to right (light green arrow).

One patient had acute myocardial infarction requiring primary angioplasty with two stents (the initial procedure). The patient was also detected to have ASD and significant left ventricular diastolic dysfunction peri-procedure. The associated left ventricle diastolic dysfunction necessitated FASD implantation (Figure 4).

The mean left-to-right shunt ratio was 2.1, and the average mean pulmonary artery pressure was 27 mmHg. One 38-year-old patient with large ASD had severe pulmonary hypertension with an elevated pulmonary vascular resistance index (PVRI) of 8.04 wu.m^2^. Two patients had moderate pulmonary hypertension. The rest of the patients had either normal pulmonary artery pressure or mild pulmonary hypertension, which improved with the closure of the ASD with the FASD occluder. This improvement was attributed to the reduction of the left-to-right shunt. The mean basal LVEDP was 16.75 mmHg. Among the adult cohort, coronary angiograms were normal except for one patient who had undergone coronary artery stenting due to double-vessel disease with good stent flow.

Balloon occlusion of the ASD was performed on eight patients with diastolic dysfunction with appropriately sized Equalizer balloons (Boston Scientific, Marlborough, MA, USA). The balloon was kept inflated for up to 15 minutes. All eight patients had an elevation of LVEDP of more than 5 mmHg from their baseline on balloon occlusion. There was no decrease in systemic pressure after balloon occlusion, and hemodynamic was maintained. The measurement of Qp:Qs was not taken after the balloon occlusion of the ASD in view of the stable hemodynamics of the patient. The patients with ASD and diastolic dysfunction were administered diuretics (loop diuretic and spironolactone) and beta-blockers before the procedure. Patients who experienced post-procedure diastolic dysfunction were maintained on spironolactone and beta-blocker therapy. Two out of eight patients with left ventricular diastolic dysfunction had mild mitral regurgitation. The regurgitation remained at the same level post-procedure.

The size of the occluders used ranged from 28 to 40 mm, with 34 mm being the most common. All patients underwent successful placement of the device. All patients except one had custom-made Lifetech fenestrated septal occluder implantation. The one patient who did not have the custom-made Lifetech fenestrated septal occluder had a handmade fenestration of an Amplatzer septal occluder. All patients received aspirin, and clopidogrel was given to four patients (three for large devices and one with a coronary stent). One patient with higher pulmonary resistance received warfarin with aspirin and a pulmonary vasodilator, and one patient had transient atrial arrhythmia during the procedure, which responded to an adenosine bolus dose. One patient on dual antiplatelet developed ecchymosis. The rashes were thought to be due to the clopidogrel, and its use was stopped. The skin rash resolved, and the patient was kept on aspirin. There were no other significant complications during the procedure or follow-up.

**Figure 5:**
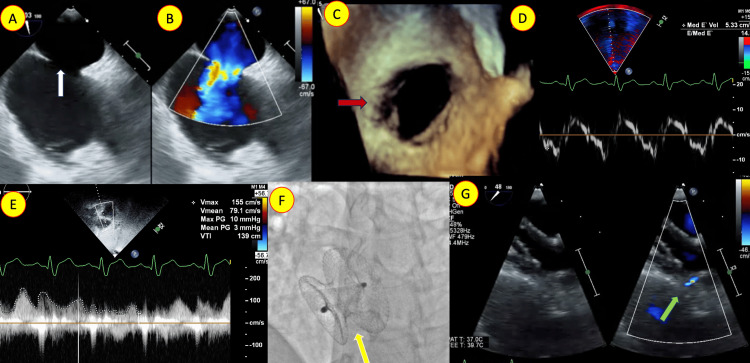
Transesophageal echocardiogram (TEE) with color Doppler (A, B) showing the moderate secundum atrial septal defect (ASD) (white arrow) left-to-right shunt. (C) Three-dimensional enface view in the TEE showing the ASD (red arrow). The diastolic dysfunction is reflected by increased E-E’ of the medial mitral annulus (D) and (E) elevated gradient across the ASD. (F) Fluoroscopic image in the oblique view shows a bulky, oversized device (yellow arrow). (G) TEE after device closure with color Doppler showing a bulky, oversized ASD device and a very small fenestration flowing left to right (green arrow).

The patients were doing well in the relatively short follow-up period (average ~6.3 months). The functional class improved in all patients. The patient with pulmonary hypertension symptoms improved from functional class III to functional class II. The patients were regularly monitored through echocardiography to assess fenestration flow, ventricular function, and the presence of pulmonary hypertension, among other factors. It is worth noting that echocardiographic assessments show a strong correlation with measurements derived from cardiac catheterization, specifically in terms of pulmonary arterial pressure and left ventricular diastolic function. The right ventricular systolic pressure (RVSP) estimated by the tricuspid regurgitation jet was normal for three patients, and eight patients had a mild elevation of the RVSP. One patient with an elevated PVRI in the pre-procedure had a moderate elevation of the RVSP in the follow-up. The diastolic function of all the patients either remained the same or improved from the previous state. Four patients had significant left ventricle diastolic dysfunction with fenestration showing a gradient between the left-to-right atrium. Other patients had mild (n = 2) or moderate (n = 2) left ventricular diastolic dysfunction determined by the echocardiographic assessment during the follow-up evaluation. One patient had a transient elevation from a mild to moderate level of pulmonary arterial pressure before discharge, which had settled at the one-month follow-up. All but one patient had flowing fenestration. One patient had reduced fenestration flow, attributed to an oversized device (Figure 5).

One patient was from a neighboring country and could only attend a physical follow-up at one year, followed by local follow-ups. On a telephone inquiry, the patient communicated about her good health and functional activity. The follow-up duration for this patient was considered as a one-year physical follow-up only.

## Discussion

The incidence of ASD is reported to be 1.64 per 1,000 live births [7]. Secundum ASDs constitute the majority (70-80%) of the ASD cohort. A left-to-right shunt at the atrial level does not produce significant symptoms early in life, leading to underdiagnosis and delayed treatment. The prolonged presence of a left-to-right shunt can result in right heart dilatation, pulmonary hypertension, atrial arrhythmia, and right ventricular dysfunction, with increased morbidity and mortality [1,8].

The management of ASD presenting with significant left-to-right shunt and accompanying comorbidities presents a clinical challenge. The introduction of FASD devices offers a valuable solution for patients where complete occlusion is unfeasible due to hemodynamic and medical reasons. This study investigated the spectrum of patients diagnosed with secundum ASD and associated comorbidities recommended for FASD closure. These findings highlight the complexity of this patient population and the potential benefits of fenestrated occlusion. The presence of various comorbidities further underscores the clinical intricacy of these cases. Notably, this study encompassed patients with left ventricular diastolic dysfunction, occasionally coexisting with pulmonary hypertension and elevated pulmonary vascular resistance with overall significant atrial level left-to-right shunt. Additionally, this study observed cases of severe pulmonary valvular stenosis, right ventricular diastolic dysfunction, SLE, and left atrial arrhythmia among the cohort. Notably, a young child diagnosed with DMD and left ventricle diastolic dysfunction was also recommended for the intervention, although it was not done due to parental choice.

The majority of the patients in this cohort consisted of patients with significant ASD and left ventricular diastolic dysfunction. Prolonged shunt lesions in ASD patients can cause left ventricular diastolic dysfunction due to septal shifting to the left and prolonged relative underfilling of the left ventricle. The septum changes due to prolonged left-to-right shunt at the atrial level can lead to bowing of the interventricular septum toward the left and under-filling of the left ventricle. The elastic stiffness of the left ventricle with age can reduce diastolic compliance of the left ventricular myocardium. In a small percentage of patients (2-3.6%), sudden complete closure of the ASD can lead to significant elevation of the LVEDP due to the manifestation of masked ventricular restriction. This elevation of the LVEDP can lead to pulmonary edema [8,9]. In patients older than 60, the risk of pulmonary edema increases to 23.6% [10]. However, closure of the atrial communication typically improves the right ventricular function and filling pressure, leading to improved left ventricular compliance and diastolic function due to ventricular interdependence [11]. To assess latent left ventricular diastolic dysfunction, a baseline evaluation of the LVEDP during the catheterization can be performed, followed by occlusion of the ASD by an appropriate balloon or with the device for 15 minutes [6]. Testing the occlusion of the ASD with a mean left atrium pressure greater than 15 mmHg or an LVEDP of more than 18-20 mmHg for 15 minutes has been suggested by Woo et al. [6]. If the pressure increases by 5 mmHg after the occlusion, the device requires fenestration and continuation of heart failure medication. High-risk candidates with elevated LVEDP require a fenestrated occluder [8,10-12]. The partial closure of the ASD with more than a 1:5 shunt helps ward off adverse cardiac and pulmonary vascular remodeling. FASD closure in suitable patients is considered a better alternative with fewer perioperative risks and is less invasive [13].

One patient in the cohort underwent a FASD closure due to a significant elevation of pulmonary vascular resistance in the presence of a left-to-right shunt (Qp:Qs >1.5). Mild-to-moderate pulmonary hypertension is relatively common in patients over the age of 50 with significant ASDs. Cherian et al. reported a 17% prevalence of a pulmonary arterial systolic pressure of more than 50 mmHg in their study cohort of untreated ASD patients [14]. After closure, the pulmonary pressure usually decreases or remains steady at mild levels; however, these patients require close and continued monitoring [15-17]. In cases of significantly increased pulmonary vascular resistance, complete occlusion of the ASD may not be possible. In such situations, closure of the defect can result in the conversion of ASD and pulmonary hypertension into idiopathic pulmonary hypertension without any shunt lesion physiology. However, a sudden elevation of pulmonary vascular resistance after the closure of the defect can compromise cardiac output by reducing adequate pulmonary venous return to the left side, leading to syncope and loss of consciousness.

A left-to-right shunt greater than 1.5 is indicative of ongoing damage to the pulmonary vasculature. The principle of FASD closure in patients with ASD and pulmonary hypertension and increased pulmonary blood flow (Qp:Qs >1.5:1) is to reduce the pulmonary blood flow significantly, thereby reducing the ongoing pulmonary vascular damage by the increased pulmonary blood flow [1]. There is no universally agreed upon cut-off of PVRI for ASD closure, but a systemic to pulmonary vascular resistance (SVR:PVR) ratio of more than 0.7 is generally considered an inoperable type of ASD. In cases with an elevated PVRI, using a fenestrated device to occlude the ASD can provide a solution that allows for some flexibility. The fenestration can decompress the elevated right heart pressure if a situation arises. A study by Kaley et al. [1] showed improvement in the symptom status. The percentage of patients in functional class III was reduced from 68% to 8%, and saturation was improved from 93% to 97%. The majority of the available studies utilized a combination of targeted medical therapy (TMT) with dual pulmonary vasodilators for at least three months, FASD closure, and continued TMT post-FASD closure. The left-to-right shunt after FASD closure is reported to be negligible (1:1). Up to 40% of patients in a study by Yan et al. had normalization of the pulmonary arterial pressure, whereas approximately 25% had a recurrence of pulmonary hypertension after the TMT was discontinued [13]. In this study, only one patient had FASD closure for an ASD and elevated PVRI, which is relatively fewer than literature reports using similar implants. The FASD device was custom-made for patients and was only recently made available on request. The reluctance of the patients with ASD and pulmonary hypertension to undergo invasive procedures is another reason for the lower number of patients in this subgroup.

Two patients in this cohort underwent balloon pulmonary valvotomy (BPV) and FASD closure in single or multiple sittings. The ASD was shunting bi-directional with desaturation in one of the patients; hence, the procedure was staged. Initially, the BPV was done, and in the follow-up, the FASD closure was performed. In the other patient, the ASD was predominantly shunting left to right with evidence of bi-directionality. The right ventricular diastolic pressure was elevated in both patients. Adults with severe valvular pulmonary stenosis will have elements of right ventricular hypertrophy and stiffness, and initially, the ASD may not shunt significantly. However, once the pulmonary stenosis is relieved, the ASD may start shunting. The persistence of right ventricular hypertrophy and diastolic dysfunction can lead to elevated right ventricular end-diastolic pressure, resulting in a bidirectional shunt [18]. In such cases, complete occlusion of the ASD in adults with a predominantly left-to-right shunt may not be advisable, and a fenestrated closure can provide a safety window. Impaired right and left ventricular mechanics and adverse interventricular interactions are reported in adolescents and young adults late after balloon valvuloplasty for isolated valvar pulmonary stenosis [19]. Right ventricular diastolic dysfunction was shown by cardiac magnetic resonance studies in patients who had balloon valvuloplasty for critical pulmonary stenosis. Right ventricular fibrosis due to persistent right ventricular outflow obstruction and pulmonary regurgitation after valvuloplasty were cited for the right ventricular dysfunction [20,21]. The use of a fenestration device can provide a hemodynamic buffer in situations where elevated atrial pressure needs to be managed while maintaining systemic outflow.

Two patients in the cohort received FASD closure because of associated SLE. One had mild pulmonary hypertension, and the second had normal pulmonary artery pressure. SLE has been associated with an increased susceptibility to pulmonary hypertension. The fenestrated ASD closure in patients with SLE and ASD was performed proactively due to the increased incidence of pulmonary hypertension in SLE patients. Research has indicated that pulmonary arterial hypertension occurs at an estimated rate of 0.5-17.5% in patients with SLE [22]. Pulmonary hypertension complicates the treatment of patients with SLE. We are unable to predict who will develop pulmonary hypertension. In the past, we did not have the option of FASD closure. A fenestration of 5-8 mm will not harm the patient but can be advantageous in the event of significant pulmonary hypertension development. The shunting through the fenestration is typically minimal. If necessary, the fenestration can also be closed with another device in the future. Therefore, in SLE patients with significant ASD shunting, fenestrated closure can be a safe approach to mitigate the potential for future problems associated with elevated pulmonary vascular resistance.

ASD is a common shunt lesion associated with atrial arrhythmias. The presence of a persistent left-to-right shunt causes right heart dilatation and leads to chamber remodeling, creating an electrical substrate that predisposes individuals to various atrial arrhythmias. Though the prevalence of atrial arrhythmias can be reduced by closing the ASD, it has been observed that even after ASD closure, these patients still have a higher risk of developing atrial arrhythmias compared with the normal population [23]. One patient with a history of left atrial arrhythmia and significant ASD was recommended for FASD closure and had the possible need for an electrophysiological study and radiofrequency ablation. The management of atrial arrhythmias after closure of the shunt lesion poses a challenge. Performing an electrophysiological study of the left atrial chamber requires a transseptal puncture, which becomes extremely challenging and risky in the presence of an atrial septal occluder. Proper planning is necessary for determining the appropriate location of the puncture and the help of other imaging modalities, such as TEE and computed tomographic angiogram [24]. Catheter ablation of left atrial arrhythmias has been reported after device closure of ASDs, with septal punctures performed under TEE guidance [24]. For patients at a high risk of developing atrial arrhythmias and are vulnerable, the presence of a fenestration in the septal occluder can be beneficial for future electrophysiological analysis and intervention. Therefore, FASD closure could potentially be considered as an option for patients at a high risk of developing future atrial arrhythmias.

One patient with DMD and left ventricle diastolic dysfunction in this study was recommended for FASD closure, although it did not happen due to parental preference. Patients with DMD are at an elevated risk for developing left ventricular dysfunction independent of the therapy [25]. DMD patients with significant left-to-right shunt caused by secundum ASD can be potentially better closed with a fenestrated atrial septal occluder for managing associated left ventricle diastolic dysfunction or future ventricular dysfunction.

Previously, fenestrations were created by hand using various techniques, like in the first patient of this study. Some authors have described using a wire and dilator to create the fenestration, followed by cutting the fabric with a surgical knife [26]. Typically, one to four fenestrations were created by hand, providing immediate relief to the patient, particularly in left ventricular restrictive physiology cases. However, hand-made fenestrations were not ideal long-term, as they tended to close, as experienced by the first patient of this study. Sometimes, the fenestration required reopening by putting a stent across it, as reported in a patient with pulmonary hypertension [26,27]. Currently, hand-made fenestrations are no longer used, and purpose-built custom-made fenestrated devices are available from different companies and are superior to hand-made fenestrations. The custom-made fenestrations come in varying sizes, depending on the size of the device, and can accommodate larger defects without compromising safety and stability. This allows for tailoring the treatment to the specific needs of a patient with a large ASD by choosing the appropriate size of the device. In some cases, the fenestration continued to shunt significantly, increasing the risk of paradoxical embolism. Man et al. suggested closure of the fenestration with another ASD device to prevent paradoxical embolism and associated left-to-right shunt [28].

The fenestrated atrial septal occluder represents a new addition to the existing transcatheter approaches for treating ASDs with comorbidities, providing a valuable alternative for eligible patients. A comprehensive clinical assessment is crucial in identifying suitable candidates for this type of closure device. There are many potential candidates for fenestrated atrial septal occluders, as recognized during this study by analyzing the patients who were recommended for and underwent FASD occluder implantation (Table 4).

**Table 4:** Potential Indications for fenestrated device closure of secundum atrial septal defect with significant left-to-right shunt and associated comorbidities.

Serial number	Potential indications
1	Atrial septal defect (ASD) concomitant with clinically significant impairment in left ventricular diastolic function
2	ASD with coexisting pulmonary arterial hypertension (PAH), manifesting as elevated pulmonary vasculature resistance, along with a notable elevation in pulmonary blood flow ratio (Qp:Qs >1.5:1)
3	ASD with simultaneous presence of left ventricular diastolic dysfunction and pulmonary arterial hypertension, along with a discernible augmentation in the pulmonary blood flow ratio (Qp:Qs >1.5:1)
4	ASD anomaly with substantial left-to-right shunt, coupled with a concurrent systemic disorder such as systemic lupus erythematosus (SLE). The SLE patients have the potential for the subsequent development of pulmonary arterial hypertension
5	ASD with notable left-to-right shunt, concomitant with the presence of Duchenne muscular dystrophy
6	ASD with severe valvular pulmonary stenosis, and right ventricular diastolic dysfunction. Fenestrated closure of ASD after balloon pulmonary valvotomy
7	ASD with associated likelihood of atrial arrhythmia of left atrial origin which may necessitate future electrophysiological intervention within the confines of the left atrium

Precise imaging analyses, including TTE and TEE, are necessary to define the anatomy and determine the appropriate size of the ASD. This will help in selecting the appropriate-sized occluder and will avoid oversizing. Additionally, concurrent lung diseases or systemic illnesses should be excluded or treated to enhance the likelihood of success by mitigating comorbidities. One elderly patient in the present study did not undergo the procedure due to significant respiratory disease and chronic renal failure. Some patients may not tolerate the hemodynamic changes resulting from ASD occlusion, even with a fenestrated occluder. These patients are not candidates for FASD occlusion. Thorough patient and family counseling regarding the nature of the intervention, the necessity of ongoing care, and the requirement for long-term medication should be emphasized. Patients and their caregivers must understand the importance of continued pulmonary vasodilator therapy in cases of pulmonary hypertension or heart failure medications for left ventricular diastolic dysfunction. This study focused on a specific group of patients with secundum ASDs who had significant comorbidities, addressed the challenges posed by these complex cases, and offered insights into managing them.

Study limitations

This study had a few limitations, including a small sample size and the fact that it was a retrospective study. The selections of patients and size of the occluders were predominantly made by a single lead operator, which could have added some bias. Additionally, the follow-up period was relatively short. Therefore, a larger prospective study would be more informative in addressing these limitations.

## Conclusions

The use of FASD devices presents a promising approach to managing patients with secundum ASDs who have additional comorbidities. This study explored the application of FASD closure for patients with significant left-to-right shunt and associated medical conditions, such as pulmonary hypertension, left ventricular diastolic dysfunction, valvar pulmonary stenosis, SLE, atrial arrhythmias, and diseases such as DMD. The findings of this study revealed that FASD closure can offer a tailored solution for patients who are not suitable candidates for complete ASD occlusion due to complex hemodynamics or medical reasons. While the study sample size was small, and limitations existed due to the retrospective design, the outcomes of this study demonstrate the potential benefits of FASD closure in carefully selected patients. However, larger prospective studies with long-term follow-ups are necessary to evaluate the safety and efficacy of the fenestrated atrial septal occluder device in suitable patients.
